# Potential modulating effects of *Allium mongolicum* regel ethanol extract on rumen fermentation and biohydrogenation bacteria of dairy cows *in vitro*

**DOI:** 10.3389/fmicb.2023.1272691

**Published:** 2023-10-30

**Authors:** XiaoYuan Wang, Chen Bai, Khas Erdene, Ashraf Muhammad Umair, QiNa Cao, ChangJin Ao, LinShu Jiang

**Affiliations:** ^1^Key Laboratory of Animal Nutrition and Feed Science in Inner Mongolia Autonomous Region Universities, College of Animal Science, Inner Mongolia Agricultural University, Hohhot, China; ^2^Beijing Key Laboratory of Dairy Cattle Nutrition, College of Animal Science and Technology, Beijing University of Agriculture, Beijing, China

**Keywords:** *Allium mongolicum* regel, rumen fermentation, rumen hydrogenation, dairy cows, *in vitro*

## Abstract

The objective of this study was to evaluate the potential modulating effects of *Allium mongolicum* regel ethanol extract (AME) on rumen fermentation and biohydrogenation (BH) bacteria *in vitro*. Four Holstein cows were used as donors for the rumen fluid used in this study. In experiment 1, five treatments (supplemented with 0 mg/g, 1 mg/g, 2 mg/g, 3 mg/g, and 4 mg/g of AME based on fermentation substrate, respectively) were conducted to evaluate the effects of different levels of AME on fermentation status *in vitro*. The results showed that after 24 h of fermentation, MCP was reduced with AME supplementation (*p* < 0.05), and the multiple combinations of different combinations index (MFAEI) value was the highest with 3 mg/g of AME. In experiment 2, six treatments were constructed which contained: control group (A1); the unsaturated fatty acid (UFA) mixture at 3% concentration (A2); the mixture of A2 and 3 mg/g of AME (A3); 3 mg/g of AME (A4); the UFA mixture at 1.5% concentration (A5); the mixture of A5 and 3 mg/g of AME (A6). The abundance of bacterial species involved in BH was measured to evaluate the potential modulating effect of AME on rumen BH *in vitro*. Compared with the A1 group, the A3, A4, and A6 groups both showed significant decreases in the abundance of rumen BH microbial flora including *Butyrivibrio proteoclasticus*, *Butyrivibrio fibrisolvens*, *Ruminococcus albus* and *Clostridium aminophilum* (*p* < 0.01). The A3 group was less inhibitory than A4 in the abundance of *B. proteoclasticus*, *B. fibrisolvens*, and *R. albus*, and the inhibitory effect of the A6 group was higher than that of A4. In conclusion, the supplementation with 3 mg/g of AME could modulate the rumen fermentation and affect BH key bacteria, which suggests that AME may have the potential to inhibit the rumen BH of dairy cows.

## 1. Introduction

Plant extracts (PE) have been extensively studied in recent ruminant research due to their significant regulatory effects on rumen fermentation (Cobellis et al., 2016) and rumen biohydrogenation (BH; Durmic et al., 2008). In addition, UFAs are of considerable benefit to human health (Du et al., 2018), and reduced intake of saturated fatty acid (SFA) products may reduce cardiovascular disease risk (Xie et al., 2018). Improving the unsaturated fatty acid (UFA) profile of meat and milk is an important topic in ruminant production. Regulating the rumen fermentation pattern and optimizing the structure of rumen microflora involved in hydrogenation have been considered effective approaches to increase the level of post-ruminal UFAs (Dewhurst and Moloney, 2013). In addition, rumen health is crucial for ensuring high-quality livestock products (Pang et al., 2022). Pan et al. (2014) reported that dietary-supplemented PE facilitates maintaining the homeostasis of the rumen environment and promotes ruminant performance. Griinari et al. (1988) reported that the rumen fermentation may cause milk fat depression, characterized by a decline in rumen pH and a shift in the rumen pattern of VFA (lower acetate and higher propionate proportions).

Partial PE supplementation in diets could modify the UFA composition of meat and milk (Frutos et al., 2020). Dietary inclusion of grapeseed, which contains high levels of phenolic compounds, resulted in greater linoleic acid (LA) and CLA levels in milk (Correddu et al., 2016). This may be related to the regulation effect of PE on rumen fermentation and BH. Diet supplementation with compound phenolic enriched *yerba mate* extracts resulted in less BH time, which increased the content of polyunsaturated fatty acid (PUFA) deposited in lamb tissues (Pena-Bermudez et al., 2022). Another study pointed out that supplementation of essential oil from anise increased ruminal pH and increased UFA concentrations in the rumen (El-Essawy et al., 2021). Furthermore, A recent study showed that condensed tannins from grapeseed induced slight change in microbial structural lipids, which decreased the level of cis-9, cis-12 C18:2 and increased the levels of cis-9, trans-11 C18:2 and trans-11 C18:1 *in vitro* (Costa et al., 2017). According to Liu et al. (2022), including *Moringa* leaf flavonoids in the diet can boost the protein content in milk. Similarly, Zhao et al. (2022a) discovered that adding citrus peel extract to milk can decrease SFA and increase MUFA and CLA due to its high flavonoid content. Santos et al. (2014) also support this finding. Kumar et al. (2014) pointed out that the possibility of PE flavonoids may improve the production performance of ruminants by changes in the rumen microbiota and intermediate nutrient metabolism. Another study showed that *Pistacia atlantica* gum essential oil decreased the production of propionic acid and increased the relative abundance of *Ruminococus flavefaciens* (Naseri et al., 2022). *Butyrivibrio* (Paillard et al., 2007; Choi et al., 2009; Maia et al., 2010; Ramos-Morales et al., 2013), *Ruminococcus albus* (Nam and Garnsworthy, 2007) and *Clostridium aminophilum* (Moon et al., 2008) have been considered to play a major role in the rumen fermentation and ruminal BH process. Buccioni et al. (2015) reported that diet supplementation with quebracho tannins decreased the abundance of *Butyrivibrio proteoclasticus* and *Butyrivibrio fibrisolvens* and increased the levels of cis-9 trans-11 conjugated linoleic acid (CLA) and vaccenic acid (VA) in milk fat. It has been shown that phenolic compounds suppressed the last step of BH of PUFAs, which was achieved by inhibiting the proliferation and activity of *Butyrivibrio proteoclasticus* (Vasta et al., 2010).

*Allium mongolicum* regel (AMR), a typical herb in the genus Allium and the family Liliaceae, grows extensively in the northern and northwestern grasslands and deserts in China (Ding et al., 2021). The extracts of AMR have been shown to regulate the rumen environment and FA metabolism by increasing the concentration of total VFA and propionic acid in the rumen and the contents of UFAs in the longissimus dorsi muscle of lambs (Liu et al., 2019). Recent research reported that C17:0, and C18:0, which correlated with the “mutton flavor” of *Small-Tailed Han* sheep were decreased by adding AME (Watkins and Frank, 2019; Zhao et al., 2022b). The structure of rumen microflora is closely related to FA deposition in the longissimus dorsi muscle. Diet supplemented with AME changed the rumen fermentation pattern and the structure of rumen microflora (Zhao et al., 2022c), thus influencing the FA metabolism by improving MUFA and PUFA deposition in longissimus dorsi muscle (Zhao et al., 2022b). Existing research has demonstrated that AMR has significantly modulating effects on rumen microbial flora and FA metabolism in mutton sheep, its potential modulating effects on the rumen of dairy cows remain unknown.

Rumen fermentation and fatty acids BH could influence the UFA metabolism in dairy cows and thus regulate milk UFA profile. It is speculated that the AME would regulate the rumen fermentation and key bacteria involved in BH in dairy cows. Thus, this experiment aimed to evaluate the effect of AME on the bacteria involved in the hydrogenation and rumen fermentation of dairy cows *in vitro.*

## 2. Materials and methods

### 2.1. AME preparation

AMR was purchased from the company (Haohai Technology Co., Ltd., Alxa League, China) and was dried at 65°C and then ground (DFT-300, Shanghai Xinnuo Instrument Group CO., LTD, Shanghai, China) to powder form, was passed through an 80-mesh sieve (Shaoxing Shangyu Shengchao Instrument CO., Zhejiang, China).

The dried AMR powder was mixed with petroleum ether (Tianjin Beilian Fine Chemical Development Co., Tianjin, China) at a ratio of 1:5 (wt/vol) and placed on a shaker (KS 501 digital, IKA (Guangzhou) Instrumentation Co., Guangdong, China) set at 100 r/min for 24 h, after which the upper layer of petroleum ether was discarded to obtain the defatted and decolored AMR powder.

After defatting and decolorization, the AMR powder was mixed with 75% ethanol at a ratio of 1:30 (wt/vol), placed in an ultrasonic cell pulverizer (KQ-300DE, Kunshan Ultrasonic Instruments Co., Ltd., Jiangsu, China) and then filtered through a vacuum pump (FUD-2110, Tokyo Rikakikai CO., LTD., Tokyo, Japan) to obtain a filtrate. The filtrate was added to 75% ethanol and mixed at a ratio of 1:30; the above process was repeated, and the resulting filtrate was distilled through a rotary evaporator (65 r/min, 75°C, 15–18 min) to obtain the AME concentrate, which was lyophilized to remove excess water and obtain the AME powder. The main components of AME are flavonoids, organic acids and their derivatives, nucleotides and their derivatives, amino acids, phenol amine, choline, lipids, and others (Zhao et al., 2021).

### 2.2. Experimental design

The experiment contained two parts:

Experiment 1 was conducted to screen appropriate AME supplementation concentrations for experiment 2. The treatments were supplemented with 0 (control group), 1, 2, 3, and 4 mg of AME per gram of fermentation substrate for simulated diets. Each treatment had three technical replicates.

Experiment 2 contained 6 treatments. The treatments were designed as rumen fluid culture supplemented with (1) none (control group, A1); (2) 3% UFA (mixture of cis-9 C18:1, cis-9,cis-12 C18:2 and cis-9,cis-12,cis-15 C18:3, A2); (3) 3% UFA plus AME (A3); (4) AME (A4); (5) 1.5% UFA (mixture of cis-9 C18:1, cis-9, cis-12 C18:2 and cis-9, cis-12, cis-15 C18:3, A5); and (6) 1.5% UFA plus AME (A6); the supplementation of AME was 3 mg/g.

Each treatment had three technical replicates. The AME supplemental concentration in experiment 2 was determined from experiment 1. The UFA mixture contained the same levels of cis-9 C18:1, cis-9, cis-12 C18:2, and cis-9, cis-12, cis-15 C18:3 and was purchased from Sigma-Aldrich [Sigma Aldrich (Shanghai) Trading Ltd., Shanghai, China].

### 2.3. Incubation with ruminal fluid *in vitro*

Experiments were conducted in December 2021. The rumen fluid was obtained from Beiya Halal Slaughterhouse in Hohhot, China (111°63′E, 40°80’N). Four Holstein cows (550 ± 30 kg, two cows for each experiment) were subjected to strict quarantine at the slaughterhouse to ensure healthy body condition. The rumen fluid sample was filtered through 4 layers of gauze and then transported in a preheated flask with CO_2_ at 40°C to prevent temperature drop before sampling. In experiment 1, rumen fluid was mixed with artificial saliva at a ratio of 1:2 (15 mL,30 mL) and added to the culture flasks that were prefilled with different concentrations of AME and fermentation substrate, for details of each additive, refer to 2.2 (Experimental design). Artificial saliva was constructed according to a previous study (Menke et al., 1979). The composition and nutrient levels of the fermentation substrate are shown in Table 1. Fermentation was performed in an air bath shaker (39°C, 120 r/min). Fermentation lasted 24 h, and the samples were immediately placed on ice at the 24th hour to ensure that fermentation was stopped simultaneously to determine fermentation indicators.

**Table 1:** Substrate composition and nutrient levels.

Items	Content (%)	Nutrients levels	Content (%)
Ingredients (%)		AME, MJ/kg	18.17
Corn	18.80	Ether extract (EE)	6.41
Soybean meal	12.22	Neutral-detergent fiber (NDF)	35.34
Cottonseed meal	2.14	Acid-detergent fiber (ADF)	36.27
Distiller’s dried grains with soluble (DDGS)	3.93	Ash	11.75
Premixes	2.14	Crude protein (CP)	15.56
Bicarb	0.51		
Fat powder	0.64		
Puffed soybeans	1.92		
Corn silage	29.09		
Flake corn	9.17		
Alfalfa	12.50		
Cotton seed	6.95		
Total	100		

Abbreviations: AME, apparent metabolizable energy.

The procedures for acquiring and cultivating rumen fluid in experiment 2 were consistent with experiment 1. The culture flasks that were prefilled with different concentration of UFA or AME and fermentation. This approach ensured the maintenance of standardized experimental conditions throughout the entire study.

### 2.4. Sample collection and analysis

At 24 h of fermentation in experiment 1 and at 24 h of fermentation in experiment 2, samples were collected to immediately determine pH by using a precalibrated hand-held precision pH meter (PHS-3C, Shanghai Osterol Industrial Co., Shanghai, China). After pH determination, the collected filtrate was placed in a centrifuge, centrifuged at 4000 r/min for 10 min, and stored at −20°C to determine ammonia-N (NH_3_-N), microbial crude protein (MCP) and volatile fatty acid (VFA) levels. Other samples were stored at room temperature overnight to count the protozoa using a hemocytometer. The rumen fluid of each sample was centrifuged, and the pellet was collected in a 5-mL cryopreservation tube and stored immediately at −80°C until DNA extraction.

The concentration of NH_3_-N was determined in the filtered solution by the indophenol colorimetric method (Chaney and Marbach, 1962). The VFAs of rumen fluid samples were determined using gas chromatography (GC-2014, SHIMADZU), which was equipped with a hydrogen flame detector and a capillary column (SHIMADZU, Technologies; 60-m long, 0.25-mm diameter; 50-μm film thickness) according to the method described in a previous study with modifications (Guo et al., 2021). The concentration of MCP was determined by the Coomassie brilliant blue method (Broderick and Craig, 1989).

The DNA of rumen bacteria was extracted by an E.Z.N. A^®^ Bacterial DNA Kit (Omega Bio-Tek, Inc., Norcross, GA, United States) according to the instructions from the manufacturer. The final elution volume was 100 μL, and the NDA concentration and purity were measured by a spectrophotometer (NanoDrop 2000c; Thermo Fisher Scientific, DE, United States). The plasmid construction was confirmed, and the primer sequences (Table 2) were designed using primer-BLAST (NCBI). Each sample contained 2 μL of DNA, 0.4 μL 50 × ROX Reference Dye1 (Biolile, Xi’an, China), 0.8 μL of each primer, and 6 μL ddH_2_O in a final volume of 20 μL. Total DNA extracted from rumen samples was used as a template for PCR amplification. Six species of bacteria involved in hydrogenation were preamplified by qPCR, and two samples failed to amplify. The PCR program included 95°C for 5 min initial denaturation, 1 PCR cycle of 95°C for 30 s, 35 PCR cycles of 58°C for 30 s, and a final extension step at 72°C for 1 min.

**Table 2:** Primer sequences.

Target species	Primer sequences (5′-3′)	Annealing temp. (°C)	Product size (bp)	The source of primer
*Butyrivibrio fibrisolvens*	F: TCAACTCCGGTATTGCATTGGA R: TCTAATCCTGTTTGCTCCCCAC	55	174	*Pseudobutyrivibrio xylanivorans* strain MA3014 chromosome 1, complete s – Nucleotide – NCBI (nih.gov)
*Butyrivibrio proteoclasticus*	F: TCCTAGTGTAGCGGTGAAATG R: TTAGCGACGGGCACTGAATGCCTAT	62	188	Clostridioides difficile strain M7404 chromosome, complete genome – Nucleotide – NCBI (nih.gov)
*Ruminococcus albus*	F: GCTCGTGTCGTGAGATGTTG R: CCACCTTCCTCCGTTTTGT	55	112	Biogenics Inc. (BGI, Inc. Beijing, China) commissioned the design of the primer.
*Clostridium aminophilum*	F: ACGGAAATTACAGAAGGAAG R: GTTTCCAAAGCAATTCCAC	57	560	Cobellis et al. (2016)

The plasmid copy number was calculated using the following formula: plasmid copy number (copies/μL) = [10^−9^ × plasmid concentration (ng/μL) × 6.02 × 10^23^]/(plasmid length×660).

The plasmid abundance was calculated from the plasmid concentration determined by a NanoDrop2000 c spectrophotometer.

### 2.5. Statistical analysis

Data were analyzed by one-way ANOVA using SAS 9.21 software. Duncan’s multiple range test was used for multiple comparisons. The significant differences were indicated by *p* < 0.05, and a statistically meaningful trend was determined as 0.05 < *p* < 0.1.

When the combination effect is greater, the reaction is more favorable to the rumen’s fermentation or the component’s use. The multiple combinations of different combinations indexes (MFAEI) is the sum of the single combination effect indexes (SFAEI) of the different combinations (treatments) and can be used to determine the fermentation status of the rumen (Yuan and Wan, 2019). The calculation is as follows.

$$\mathrm{SFAEI}=\frac{\Sigma_{m}^{n}\left(T-Q\right)/n}{R}$$

m: Time of each fermentation point.

n: Total number of fermentation time points.

Q: Values of each indicator at each time point for the control group.

T: Values of each indicator at each time point for each experimental group.

R: Mean of the sum of T at each time point.

## 3. Results

The results are presented in two sections: screening suitable concentrations of AME and additives (Experiment 1), and evaluating changes in BH key bacteria *in vitro* (Experiment 2).

### 3.1. AME on rumen fermentation (experiment 1)

The effects of AME on rumen fermentation *in vitro* are outlined in Table 3. After 24 h of fermentation, the addition of AME did not affect rumen pH, TVFA, acetate, propionate, protozoa, and NH_3_-N, and MCP was reduced in response to AME supplementation (*p* < 0.05).

**Table 3:** Effect of AME at different concentrations on rumen fermentation parameters in experiment 1.

Ruminal fermentation parameters	AME concentration					SEM	*P*
	0 mg/g	1 mg/g	2 mg/g	3 mg/g	4 mg/g		
Total VFA (mmol/L)	36.69	37.22	38.03	38.82	38.35	2.69	0.87
Acetate (mmol/L)	22.01	22.28	23.11	23.48	23.07	0.95	0.79
Propionate (mmol/L)	7.63	7.46	7.56	7.68	7.53	0.32	0.99
Butyrate (mmol/L)	5.52	5.97	5.83	6.04	6.25	0.31	0.57
Valerate (mmol/L)	0.62	0.64	0.62	0.61	0.62	0.03	0.98
Isobutyrate (mmol/L)	0.33	0.31	0.33	0.42	0.31	0.03	0.06
Isovalerate (mmol/L)	0.58	0.57	0.59	0.59	0.57	0.03	0.99
Acetate: Propionate ratio (mmol/L)	2.89	2.91	3.06	3.07	3.07	0.06	0.10
pH	5.82	5.52	5.63	5.67	5.62	0.06	0.06
Protozoa (*10^4^)	5.33	5.50	5.00	4.67	4.67	0.29	0.22
Microprotein (mg/100 mL)	27.51^A^	24.09^B^	16.75^E^	21.30^C^	19.29^D^	0.36	<0.01
Ammonia-N (mg/100 mL)	57.60	47.77	55.60	48.44	51.34	2.73	0.11

The presence of the same superscript letter indicates a nonsignificant difference (*p* > 0.05). Significant differences (*p* < 0.05) are indicated if the superscript letters are different. SEM, standard error of the means.

Table 4 shows the effect of AME on rumen fermentation. The MFAEI value under an AME concentration of 3 mg/g was the absolute maximum value of the experiments in this study, and the result shows that the suitable concentration of AME in modulating rumen fermentation was 3 mg/g.

**Table 4:** SFAEI and MFAEI values under different AME concentrations in experiment 1.

Factor (SFAEI)	AME concentration			
	1 mg/g	2 mg/g	3 mg/g	4 mg/g
pH	−0.0126	0.0000	−0.0100	−0.0064
MCP	−0.0355	0.0188	−0.0655	0.0399
Ammonia-N	0.0413	−0.0280	0.0259	0.0048
Acetate	0.0673	0.1891	0.1753	0.1653
Propionate	0.0073	0.0956	0.0925	0.0898
A: P	0.0349	0.0839	0.0724	−0.0181
TVFA	−0.0448	0.0551	0.0446	0.0309
Protozoa	−0.1400	−0.0074	−0.0917	−0.0398
MFAEI	0.2664	−0.0819	0.4070	0.2434

### 3.2. AME-USFA and AME on rumen fermentation (experiment 2)

The effects of AME and UFA on rumen fermentation in experiment 2 are presented in Table 5. There were no effects of additives on rumen concentrations of acetate and total VFA. Whereas the amount of Propionate in the A4 group was lower than in other groups (*p* < 0.01), and the proportion of Acetate: Propionate ratio in the A4 group was higher than in other groups (*p* < 0.01). Significantly lower concentrations were detected in Butyrate acid that was supplemented with both UFA and AME groups (*p <* 0.01). The pH of A1 was significantly lower than that of the other treatment groups (*p* < 0.01). The number of protozoa in the A5 and A6 groups was significantly lower than that in the A1, A2, A3, and A4 groups. In addition, the number of protozoa in the A5 group was significantly lower than that in the control group and the other treatment groups (*p* < 0.05). The highest concentration of MCP was observed in the A3 group (*p* < 0.01), and the A4 group was significantly lower than the control group and other treatment groups (*p* < 0.01). The concentrations of NH_3_-N in the A1, A2, and A4 groups were significantly lower than those in the A3, A5, and A6 groups (*p* < 0.01).

**Table 5:** Effects of AME on rumen fermentation in experiment 2.

Metric (24 h)	Groups						SEM	*P*
	A1	A2	A3	A4	A5	A6		
Total VFA (mmol/L)	79.94	75.24	79.28	72.54	79.28	85.27	2.48	0.05
Acetate (mmol/L)	45.61	40.04	43.48	42.68	45.07	49.32	1.99	0.09
Propionate (mmol/L)	26.37^AB^	30.39^A^	29.82^A^	22.38^B^	27.00^A^	28.17^A^	1.41	0.02
Butyrate (mmol/L)	6.17^A^	3.50^C^	4.55^BC^	5.70^A^	5.35^AB^	5.86^A^	0.35	<0.01
Valerate (mmol/L)	0.78	0.62	0.74	0.77	0.86	0.92	0.07	0.16
Isobutyrate (mmol/L)	0.43	0.35	0.47	0.44	0.42	0.54	0.03	0.13
Isovalerate (mmol/L)	0.78	0.62	0.74	0.77	0.86	0.92	0.07	0.16
Acetate: Propionate ratio (mmol/L)	1.76^AB^	1.32^C^	1.48^BC^	1.91^A^	1.67^ABC^	1.75^AB^	0.12	0.04
pH	6.30^C^	6.44^AB^	6.36^BC^	6.50^A^	6.53^A^	6.51^A^	0.04	<0.01
Protozoa (*10^4^)	8.67^A^	8.00^AB^	9.33^A^	8.33^AB^	5.33^C^	6.33^BC^	0.69	<0.05
Microprotein (mg/100 mL)	1.23^C^	1.46^A^	1.52^A^	1.18^C^	1.43^AB^	1.33^B^	0.03	<0.01
Ammonia-N (mg/100 mL)	24.37^A^	23.64^B^	27.04^A^	23.84^B^	26.72^A^	26.88^A^	0.58	<0.01

A1 is the control group; A2 represents the UFA mixture of oleic acid (C18:1), linoleic acid (C18:2) and linolenic acid (C18:3), and 1% of each UFA was added to the substrate, totally 3%; A3 represents a mixture of A2 and AME; A4 represents the addition of AME only; A5 represents the UFA mixture of oleic acid (C18:1), linoleic acid (C18:2) and linolenic acid (C18:3), and 0.5% of each UFA was added to substrate, totally 1.5%; A6 represents a mixture of A5 and AME.The presence of the same superscript letter indicates a nonsignificant difference (*p* > 0.05). Significant differences (*p* < 0.05) are indicated if the superscript letters are different.SEM, standard error of the means.

### 3.3. AME-USFA on rumen biohydrogenation-related microbial flora (experiment 2)

Supplementation with AME and the AME-UFA mixture significantly affected the bacteria involved in hydrogenation, as shown in Table 6. Compared with the control group, the treatment groups exhibited decreased relative abundances of *B. proteoclasticus*, *R. albus,* and *B. fibrisolven* (*p* < 0.01; Table 5). The abundance of *B. proteoclasticus* in A2 was significantly lower than that in A5 (*p* < 0.01). The A4, A5, and A6 groups showed inhibition of *R. albus* growth, and *R. albus* growth in the A6 group was significantly lower than that in the other treatment groups (*p* < 0.01). Supplementation with AME and UFAs also significantly affected the relative abundances of *B. fibrisolvens*, and the abundance of *B. fibrisolvens* was lower than that in the control group in each treatment (*p* < 0.01). The growth of *C. aminophilum* was inhibited (*p* < 0.01) in the AME supplementation group (A4) after fermentation for 24 h.

## 4. Discussion

### 4.1. Effects of AME on rumen fermentation

The effects of PE on ruminal fermentation are variable (Naseri et al., 2022). In experiment 1, the concentration of NH_3_-N in the rumen fluid did not change in the AME supplementation group at 24 h. It has been reported that PE may not affect the concentration of NH_3_-N (Kozłowska et al., 2021). Moreover, a previous study reported that tannins as feed additives to modulate ruminal fermentation and ruminal BH in dairy ewes had no effects on NH_3_-N concentration (Toral et al., 2011). The addition of garlic oil to the diet of lactating dairy cows did not affect the concentration of NH_3_-N (Blanch et al., 2016). Furthermore, the NH_3_-N concentration in dairy cows did not change when feeding them high or low doses of *Origanum vulgare L.* extract (Olijhoek et al., 2019). However, a previous study reported that *Pistacia atlantica gum* essential oil may change the ruminal NH_3_-N concentration (Naseri et al., 2022). In addition, a high dose of thymol decreased the ruminal concentration of NH_3_-N, but lower doses did not affect the NH_3_-N concentration (Castillejos et al., 2006). Vakili et al. (2013) indicated that the effect of thymol on ruminal NH_3_-N concentration is dose-dependent. Zhao et al. (2022a) pointed out that AME supplementation in lambs did not affect ruminal NH_3_-N. In addition, although the treatments did not significantly affect the NH_3_-N concentration, 4 mg/g AME supplementation increased the *in vitro* MCP concentration after 24 h of fermentation.

A previous study considered protozoan variation to be positively correlated with NH_3_-N concentration (Spanghero et al., 2022). However, in another study, the type and concentration of the main active compounds in different PEs resulted in inconsistent protozoan population results (Soltan et al., 2018). In the present study, the number of protozoa was decreased by AME supplementation at 24 h. A study by Totakul et al. (2022) suggested that *Chaya* may directly inhibit the protozoal population by interrupting cell wall synthesis or nucleic acid synthesis. The additional microbial reduction caused by PE may be attributed to the destruction of the cell membrane. A study by Francis et al. (2002) showed that *phyto-saponins* can form irreversible complexes with cholesterol in the bacterial cell membrane, which causes membrane breakdown and cell death. However, research by Śliwiński et al. (2002) demonstrated that saponin-rich products did not affect rumen fluid protozoal counts. Another study suggested that the type and origin of the PE may play a role in the differential effects against protozoa (Makkar and Becker, 1996).

Some previous studies have found that the effects of PE supplementation on rumen fermentation may be contradictory. It has been reported that flaxseed oil supplementation did not affect VFAs (Ueda et al., 2003). In another major study, Yang et al. (2022) found that adding flaxseed oil did not shift the concentration of VFAs, in the meantime, UFA proportion would change the microbial community’s diversity and abundance. Our study in experiment 2 found that adding AME can significantly decrease the concentration of MCP and increase the concentration of NH_3_-N, which indicated that AME may regulate rumen fermentation by changing the microflora. Additionally, we noticed that the pH was significantly increased in A4 as compared with the A1 group, which might affect the rumen BH pathways by AME supplementation. Harvatine et al. (2009) opined that typical pathways of rumen BH are altered when rumen pH is low. The low proportion of cis-9, trans-11 CLA, and trans-11 C18:1 at low pH was also observed by Troegeler-Meynadier et al. (2003). Piperova et al. (2002) observed that the low rumen pH resulted in higher trans-10 C18:1 produced and the effluent proportion of trans-10 C18:1 was positively related to BH (Bauman and Griinari, 2003). Results of the present experiment indicate that the pH is an important factor that may affect the BH process. It appears that a substantial correlation exists between AME and pH in relation to rumen BH. A possible hypothesis is that higher pH levels lead to a decrease in hydrogen ions, which are essential for hydrogenation during the process. Alternatively, an increase in pH induced by AME supplementation may potentially affect the hydrogenation process by limiting the availability of hydrogen ions in the rumen. However, additional experiments are needed to verify this hypothesis.

### 4.2. Effects of AME on rumen biohydrogenation bacteria

PE may have the potential to limit ruminal BH (Guerreiro et al., 2016). Published research suggests that PE causes certain changes in the rumen microflora and beneficial changes in rumen lipid metabolism (Huws et al., 2013). The inhibitory effect of PE on hydrogenation has been reported before. A study by Wood et al. (2010) suggested that PE potentially inhibits BH by acting on microorganisms. Several bacteria involved in BH function were identified: *B. fibrisolvens*, *B. proteoclasticus*, and *B. crossotus* (Palevich et al., 2017). Moreover, other microorganisms may participate in the process of BH, such as members of the *B. fibrisolvens* group, which convert LA to VA (trans-11-18:1) via cis-9, trans-11-CLA (Ramos-Morales et al., 2016).

**Table 6:** Effects of AME on key bacteria of rumen hydrogenation in experiment 2 [lg (copies/μL)].

Metric (24 h)	Groups						SEM	*P*
	A1	A2	A3	A4	A5	A6		
*Butyrivibrio proteoclasticus*	4.71^A^	1.49^C^	2.22^BC^	2.20^BC^	2.45^B^	2.17^BC^	0.26	<0.01
*Ruminococcus* *albus*	3.76^A^	1.28^BCD^	1.47^BC^	1.81^B^	0.88^CD^	0.71^D^	0.21	<0.01
*Butyrivibrio fibrisolvens*	3.44^A^	0.53^C^	0.84^BC^	1.55^B^	0.87^BC^	0.76^C^	0.24	<0.01
*Clostridium aminophilum*	0.20^BC^	0.23^AB^	0.28^A^	0.08^E^	0.13^DE^	0.15^CD^	0.02	<0.01

The presence of the same superscript letter indicates a nonsignificant difference (*p* > 0.05). Significant differences (*p* < 0.05) are indicated if the superscript letters are different.

Diet supplementation with UFAs may interfere with the BH process (Kamel et al., 2018). A study by Maia et al. (2010) suggested that UFAs play a preventive role in the BH process and disrupt the lipid bilayer structure of bacteria. Studies of other sulfur-containing PEs have also shown inhibitory effects on gram-positive bacteria. Busquet et al. (2005) suggested that the inhibitory effect of garlic oil may be mediated by inhibiting HMG-CoA reductase, which plays a role in the synthesis of isoprenoid ethers, and this process is also achieved by mediating the cell membrane (Busquet et al., 2005). A previous study showed that phenolic compounds have a toxic effect on certain rumen microbes, which contributed to the permeability of membranes and inhibited the enzyme activity of ruminal microorganisms (Jones et al., 1994). Moreover, research has shown that phenolic compounds can trigger endogenous oxidative stress in bacterial cells by inducing ROS formation, which can lead to bacteria cell death (Efenberger-Szmechtyk et al., 2021).

Kim et al. (2015) showed that rumen fluid incubated with PE (*Punica granatum, Betula schmidtii, Ginkgo biloba,* and *Camellia japonica*) decreased populations of *R. albus*, which is a cellulose-digesting species that has been identified as the major hydrogenating bacteria in the rumen. These bacteria benefit fungi by rapidly hydrogenating UFAs (Nam and Garnsworthy, 2007). *R. albus* belongs to “Type A” bacteria, which can hydrogenate C18:2n-6 and C18:3n-3, with VA being their major end product (Ishlak et al., 2015). In experiment 2, AME was added at 3 mg/g as determined by experiment 1. We observed that the abundance of *R. albus* was decreased in the AME treatment groups (A3, A4, A6). Interestingly, we noticed variability in the antibacterial rate between groups treated with different concentrations of the AME-UFA mixture. The low-concentration AME-UFA mixture group (A6) exhibited a higher antibacterial rate than the low-concentration UFA group (A5), but the high-concentration UFA mixture group (A2) was not as effective as the high-concentration AME-UFA group (A3). This observation suggests that AME may play an ‘adjuvant’ role in the inhibition of *R. albus* by supplementing UFAs.

*B. proteoclasticus* is a proteolytic bacterium in the rumen that produces rumenic acid (RA) together with VA and is the only species to form stearic acid (SA; Wallace et al., 2006). Another study reported that *B. proteoclasticus* could reduce trans-18:1 FA to SA (Ramos-Morales et al., 2013). Furthermore, several studies have also verified the association between SA and decreased *B. proteoclasticus* abundance (Zhu et al., 2012). A study by Henderson (1973) noted that *Butyribrio* sp. was most sensitive to FA. Therefore, the mixed acid used in the present study was greatly toxic to *Butyribrio* sp. Although the abundance of *B. proteoclasticus* in the A4 treatment group was not different from that in the A2, A3, A5, and A6 treatment groups, it was still significantly lower than that in the A1. In addition, the antibacterial effect of AME on *B. fibrisolvens* was observed. *B. fibrisolvens* are highly prevalent gram-positive bacteria in the rumen (Maia et al., 2010). Studies have shown that *B. fibrisolvens* considerably contributes to the BH of UFAs in the rumen. In our study, the antimicrobial effect of UFAs at high concentrations (A2) was more pronounced than that of the AME-UFA mixture (A3, A6), and the antimicrobial effect of the AME-FA mixture (A3, A6) was more pronounced than that of the single AME additive (A4). Despite this, the A4 group significantly inhibited (*p* < 0.01) the growth of *B. fibrisolvens*, which indicates the inhibitory potential of AME for BH in the rumen.

*C. aminophilum* is the typical ammonia producer in the rumen (Shen et al., 2017), which is closely related to *B. proteoclasticus* and involved in BH in the rumen (Moon et al., 2008). Compared to other bacteria in this study, *C. aminophilum* was more sensitive to the A4 treatment group and low concentrations of UFAs (A5) and AME-UFA mixtures (A3, A6). Compared to the A3, A5, and A6 treatment groups, the A4 treatment group significantly inhibited the growth of *C. aminophilum* (*p* < 0.01); however, *C. aminophilum* does not appear to be largely sensitive to UFAs at high concentrations (A2), and between A1 and A2 groups, there was no significant difference in microbial counts.

AME had a significant inhibitory (*p* < 0.01) effect on *B. proteoclasticus*, and the higher concentration AME-UFA mixture group (A3) was less inhibitory than the single AME treatment group (A4). However, the inhibitory effect of the lower concentration AME-UFA mixture (A6) was higher than that of the single AME treatment group (A4), and the underlying mechanism for this phenomenon is not yet clear. Above all, AME reduced the abundance of key BH bacteria. Such impacts may contribute to the production of high-quality livestock products.

## 5. Conclusion

Supplementation of AME could modulate ruminal fermentation *in vitro* of dairy cows. The suitable concentration of AME in modulating rumen fermentation is 3 mg/g, which reduces the MCP concentration significantly without compromising total VFA and tends to reduce the concentration of propionate and the population of protozoa. AME and AME with low concentration UFA mixture decreased the abundance of *B. proteoclasticus*, *R. albus*, *B. fibrisolvens,* and *C. aminophilum*. Based on our results, AME has the potential to inhibit rumen BH and thus may influence the UFA metabolism in dairy cows. However, the results need further *in vivo* research to confirm.

## Data availability statement

The raw data supporting the conclusions of this article will be made available by the authors, without undue reservation.

## Ethics statement

The animal studies were approved by Animal Ethics Committee of Inner Mongolia Agricultural University. The studies were conducted in accordance with the local legislation and institutional requirements. Written informed consent was obtained from the owners for the participation of their animals in this study.

## Author contributions

XW: Data curation, Investigation, Methodology, Validation, Writing – original draft, Writing – review & editing. CB: Funding acquisition, Investigation, Methodology, Project administration, Software, Supervision, Writing – review & editing. KE: Supervision, Writing – review & editing. AU: Methodology, Project administration, Supervision, Writing – review & editing. QC: Software, Writing – review & editing. CA: Conceptualization, Funding acquisition, Methodology, Project administration, Resources, Supervision, Validation, Writing – review & editing. LJ: Formal analysis, Funding acquisition, Methodology, Project administration, Resources, Supervision, Validation, Writing – review & editing.
